# Human Exposure Assessment in Air Pollution Systems

**DOI:** 10.1100/tsw.2002.119

**Published:** 2002-02-23

**Authors:** Junfeng (Jim) Zhang, Paul J. Lioy

**Affiliations:** Environmental and Occupational Health Sciences Institute, University of Medicine and Dentistry of New Jersey and Rutgers University, 170 Frelinghuysen Road, Piscataway, NJ 08854,

**Keywords:** air pollution, environmental epidemiology, exposure assessment, indoor air, risk assessment

## Abstract

The air pollution problem can be depicted as a system consisting of several basic components: source, concentration, exposure, dose, and adverse effects. Exposure, the contact between an agent (e.g., an air pollutant) and a target (e.g., a human respiratory tract), is the key to linking the pollution source and health effects. Human exposure to air pollutants depends on exposure concentration and exposure duration. Exposure concentration is the concentration of a pollutant at a contact boundary, which usually refers to the human breathing zone. However, ambient concentrations of regulated pollutants at monitoring sites have been measured in practice to represent actual exposure. This can be a valid practice if the pollutants are ones that are predominantly generated outdoors and if the monitoring sites are appropriately selected to reflect where people are. Results from many exposure studies indicate that people are very likely to receive the greatest exposure to many toxic air pollutants not outside but inside places such as homes, offices, and automobiles. For many of these pollutants, major sources of exposure can be quite different from major sources of emission. This is because a large emission source can have a very small value of exposure effectiveness, i.e., the fraction of pollutant released from a source that actually reaches the human breathing zone. Exposure data are crucial to risk management decisions for setting priorities, selecting cost-effective approaches to preventing or reducing risks, and evaluating risk mitigation efforts. Measurement or estimate of exposure is essential but often inadequately addressed in environmental epidemiologic studies. Exposure can be quantified using direct or indirect measurement methods, depending upon the purpose of exposure assessment and the availability of relevant data. The rapidly developing battery and electronic technologies as well as advancements in molecular biology are expected to accelerate the improvement of current methods and the development of new methods for future exposure assessment.

## INTRODUCTION

Although air pollution is a well-known term in modern societies, precisely defining what air pollution is may not be an easy task. Historically, air pollution has been defined based on visible emission sources and has been characterized as smog episodes, which are associated with reduced atmospheric visibility and increased mortality and morbidity rates. For example, during the episode of December 1952 in London, the dense smog resulted in the concurrent buildup of particulate matter and sulfur oxide species in the atmosphere. The levels of British smoke (indicator of particulate matter, measured by the then-standard British method) and sulfur dioxide exceeded 1,000 µg/m$^3$, and there were 1,000 excess deaths per day. This type of pollution, called sulfurous or London smog, has disappeared in London and other cities in industrially developed countries as various clean air legislations led to the marked decrease in, or the elimination of, the use of dirty coal. However, the legislations may have switched pollution scale from local to regional because of the increased consumption of electricity generated in coal-fired power plants with tall stacks for effective dispersion. The situation is different in cities in developing countries, where uncontrolled combustion still occurs on both domestic and industrial scales[1,2,3]. For example, the 95$^{th}$ percentile values of total suspended particles (TSP) daily average ranged from 493 to 1,280 µg/m$^3$ in four Chinese cities during 1993–1996[4]. The highest concentrations, observed during winter months in these cities, were similar to levels of particulate matter (PM) during those London smog episodes of the 1950s, although the chemical compositions of PM may have been different[4,5,6]. The other type of smog, photochemical smog or Los Angeles smog, was first hypothesized as a component of the air pollution problem in Los Angeles in 1954[7]. Photochemical smog is formed via complex chemical reactions of hydrocarbons and oxides of nitrogen in the presence of solar radiation. Secondary pollutants of photochemical reactions include ozone and other oxidants, e.g., peroxyacetyl nitrate (PAN), sulfuric acid, nitric acid, and secondary particles of sulfate, nitrate, and oxidized organics. The mixture of these pollutants has been associated with excess morbidity and mortality and resulted in the hazy appearance of the atmosphere during the historical smog episodes in Los Angeles. Automobile emissions of the precursors were mainly responsible for the photochemical smog observed in Los Angeles[2]. As the number of automobiles increased around the world, photochemical smog eventually began to appear in other metropolitan areas. In the U.S., the expansion of the national highway systems and extensive use of the automobile also expanded residential and commercial districts into what were rural areas, which are now categorized as the suburbs. The ubiquitous nature of the ozone problem has led to an estimated >50,000,000 people exposed to ozone above the current U.S. national standard during the summer[8]. The rapid growth in automobile numbers in developing countries has already and will continue to lead to similar problems (e.g., high ozone concentrations have been reported in large metropolitan areas of Beijing, Mexico City, and Bangkok). The scale of photochemical smog is not limited to urban cores but spreads throughout larger areas. It has been documented that transport processes allow ozone and fine particles ($PM_{2.5}$) to travel great distances (i.e., >1,000s of kilometers) away from the source of their precursors[1,8].

As heavily dense and very localized smog is no longer the typical characteristic of air pollution, it is not adequate and practical to define air pollution using the hazy or smoky appearance of the atmosphere. In general, the concept of pollution entails a sense of degradation, a loss of quality, and adverse effects. In his 1975 book, Seinfeld defined air pollution "as any atmospheric condition in which substances are present at concentrations high enough above their normal ambient levels to produce a measurable effect on man, animals, vegetation, or materials"[9]. This definition elucidates the condition of polluted atmosphere. In contrast, Godish emphasizes activities/actions resulting in air pollution in his definition: "The gaseous mixture [of the atmosphere] becomes polluted when it is changed by the addition (or theoretically, subtraction) of particles, gaseous, or energy forms (e.g., heat, radiation, or noise) so that the altered atmosphere poses some harm because of its impact on weather, climate, human health, animals, vegetation, or materials"[10]. In both definitions, the judging role of effects/impacts (on human and ecologic health) is clearly presented. Hence, it appears logical to assess its impacts in order to define an air pollution problem.

Both definitions shown above reflect the historical concerns about air pollution, i.e., those that have been associated with the atmospheric (outdoor) environment. However, severe air pollution can occur in constructed environments such as occupational settings, residences, and office buildings, and as a consequence of personal habits such as tobacco smoking. Air pollution occurring in constructed environments is often known as indoor air pollution, in contrast to conventional atmospheric pollution being outdoor air pollution. In this paper, air pollution is used as a generic term; it can be outdoor air pollution, indoor air pollution, or both.

From the pollution source to potential adverse effects, the air pollution problem can be simply depicted as a system (continuum) consisting of several basic components, as illustrated in Fig. 1, where the concern of adverse effects is specifically focused on human health. Given that air pollution is virtually defined based on whether there are measurable harmful effects, let us discuss the continuum starting from health effects and go backwards. Health effects of air pollution depend on both dose received by an individual (host) and the susceptibility of the host. The host susceptibility to pollutant effects is influenced by many factors such as age, gender, ethnicity, and health conditions. Dose is the amount of substance received at a target organ or tissue. Because dose depends not only on exposure but also on host-specific parameters such as respiratory rate, adsorption/absorption rate, and metabolic rate, it is difficult to determine dose for large numbers of people. Therefore, air pollution studies have tended to focus on exposure that is usually assumed to be closely proportional to dose. Although exposure, the contact between a pollutant and a target, depends on exposure concentration and exposure duration (to be discussed in the next section), concentration alone is often used in ambient air pollution regulations to represent exposure. This has been done, for example, by measuring ambient concentrations of regulated pollutants at monitoring sites in urban areas with the assumption that the measured ambient concentrations can represent actual human exposures. The validity of this assumption, however, has been questioned by numerous exposure studies and indoor air studies for many pollutants[11,12,13,14]. Ambient concentration of a pollutant depends on its source strength and its fate after its release from the source. The environmental fate can be complex, involving physical and chemical processes such as dilution, dry or wet deposition, and chemical reactions. At the source, air pollutants can be emitted from natural processes (e.g., volcanic activity, pollen, and mold spores) or anthropogenic activities (e.g., power plant emissions, waste incineration). Secondary pollutants are generated from chemical reactions of their precursors in the atmosphere.

Described above is a direct pathway of the continuum from a pollutant source to human health effects. In this case, the air pollutant produces health effects upon an individual's inhalation of the pollutant. Some pollutants released into the atmosphere first adversely impact the climate or ecosystems (environment). The impaired environment ultimately will cause adverse health effects in humans. This indirect pathway of the continuum is shown by the arrows on the top of the boxes in Fig. 1. For example, breathing carbon dioxide and methane at typical ambient levels will not result in any health concerns. Global warming or climate change resulting from even a slight increase in global atmospheric levels of these greenhouse gases can be associated with an enormous amount of health risks[15].

## CONCEPT OF HUMAN EXPOSURE

A primary goal of air pollution regulatory programs is to protect public health from the adverse effects of pollutants. It is necessary to understand all the components of the air pollution system described as a continuum in Fig. 1 in order to determine the health risks posed by the pollutant or the pollution source. Historically, however, the component that has received the least attention is

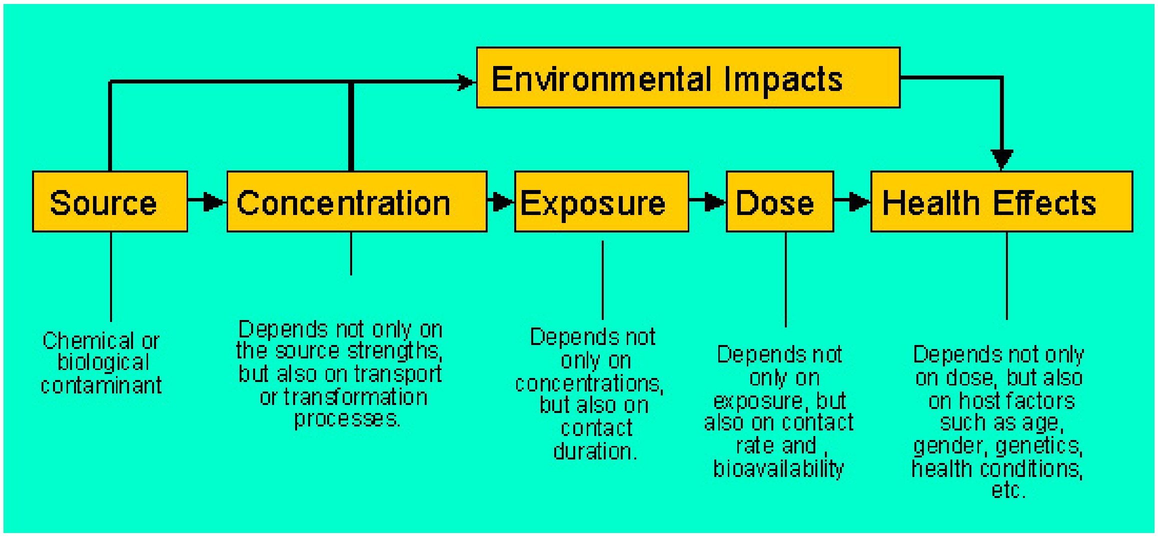

**FIGURE 1.** Air pollution system: conceptualized continuum from source to human health effects. (Adapted from [14], [20], [30].)

exposure or dose (these two terms are often used interchangeably). In fact, the term *exposure* has been often defined or used in different and sometimes confusing ways in the existing environmental health literature, although the term may describe different processes across different fields of environmental health sciences, such as exposure assessment, risk assessment, industrial hygiene, environmental toxicology, environmental epidemiology, and public health. Having reviewed various previous definitions[16,17,18,19,20,21,22,23,24,25,26,27], Zartarian et al. proposed a unified theoretical framework that included previous scientific definitions of exposure and related concepts[28]. In their theoretical framework, Zartarian et al. have defined exposure, in a generic form, as the contact between an agent (e.g., a pollutant) and a target (e.g., a human lung)[28]. The authors have presented a concept of "instantaneous point exposure" being the joint occurrence of two events: (1) at point $i$ of a target is located at $(x_i,y_i,z_i)$ at time $t$, and (2) an agent of concentration $C_i$ is present at location $(x_i,y_i,z_i)$ at time $t$. They have also shown that the definition of instantaneous point exposure is fundamental in that all other functions of exposure with respect to time and space (e.g., average exposure, integrated exposure) can be derived from it[28].

For inhalation exposure, it is necessary to know exposure over a time interval of concern, because people will normally breathe continuously the air containing pollutant of concentration $C_i$ at location $(x_i,y_i,z_i)$ during the contact period ($t_1$ to $t_2$). The mathematical expressions of the integrated exposure over the time interval of concern may be complicated depending on the spatial and temporal variations of pollutant concentration. One of most commonly used exposure definitions is *temporally integrated exposure* ($E$) as defined in the paper of Zartarian et al.[28]. Mathematically, this type of exposure is the integral (or summation for the discrete form) of instantaneous point exposure values over the time period ($t_1$ to $t_2$) (Eq. 1). This is the type of exposure primarily emphasized by the National Academy of Sciences and in the U.S. Environmental Protection Agency's exposure assessment guidelines[17,18]. Since practical measurements usually provide incremental data on exposure, time-average exposure $E_{twa}$ (e.g., time-weighted averages measured in occupational settings) is often used, which is the temporally integrated exposure divided by the duration of the time interval (Eq. 2)[17,29].

$$E = \int_{t_1}^{t_2} C(t)dt \qquad (1)$$

$$E_{twa} = \frac{\int_{t_1}^{t_2} C(t)dt}{t_2 - t_1} \tag{2}$$

Human exposure assessment can be considered as the science that describes how an individual or population comes in contact with a contaminant, including quantification of that amount across space and time, for individuals and populations[20,21]. Exposure analysis is closely related to the following science disciplines: traditional environmental science that deals with sources and environmental fate (transport and transformation) of pollutants; toxicology that deals with toxicologic mechanisms (accumulation, transformation, and elimination) of pollutants within the human body; and environmental epidemiology that examines associations between pollution exposure and health outcomes[30].

## SOURCES OF EMISSION VS. SOURCES OF EXPOSURE

The importance of exposure assessment can be easily seen from the continuum shown in Fig. 1, as it is a key to linking the pollution source and health effects. Although "dose makes poison" has been well known for centuries, the importance of exposure assessment in air pollution history has not been recognized until about 2 decades ago and is still being underestimated. Historically, air pollution has been measured at places such as the roofs of public buildings because these places are easy to access and control, but they do not necessarily reflect where the pollution is or where the people are. From the 1980s to 1990 the U.S. Environmental Protection Agency (EPA) conducted Total Exposure Assessment Methodology (TEAM) studies on volatile organic compounds (VOCs), carbon monoxide, and later on PM as well. These studies were carried out in more than a dozen states in the U.S., using thousands of carefully chosen subjects representative of most North Americans living in urban and suburban areas[31]. The results from TEAM studies were striking: people were very likely to receive the greatest exposure to many of the toxic chemicals measured in the studies not outside but inside the places they usually consider to be unpolluted, such as homes, offices, and automobiles. Even in two New Jersey cities, Bayonne and Elizabeth, both of which have chemical processing plants and busy automobile traffic, the levels of 11 VOCs proved much higher indoors than outdoors[22]. The chief sources appeared to be ordinary consumer products (e.g., air fresheners and cleaning products), building materials, and environmental tobacco smoke. Although these sources are individually small in terms of their emissions, their contribution to total human exposure can exceed the contribution of those large emission sources (e.g., Superfund sites, local industry, power plants) normally targeted by environmental regulations. For example, when considering the amount of benzene released into the general environment in the U.S., one would find from the TEAM study that the largest share comes from motor vehicles (83%), followed by industry (14%) and domestic sources (3%), and 0.1% of the total from smoking cigarettes[11]. When benzene exposure is considered, however, the picture is very different. Wallace estimated that some 45% of the total exposure of the U.S. population to benzene comes from active or passive smoking, about 36% from inhaling gasoline fumes, 16% from domestic sources (e.g., paints, gasoline stored in basements or garages), and only 3% from industrial pollution[11].

Another powerful example is the analysis conducted by Smith in comparing PM emissions and exposure between coal-fired power plants and tobacco smoke[14]. In 1990, about 0.4 Mt of PM, or 1.6 kg per person, was emitted annually from all coal-fired power plants in the U.S. Only

**TABLE 1**
**Major Emission Sources vs. Exposure Sources of Air Pollutants**

| Pollutant | Major Emission Sources | Major Exposure Sources |
|---|---|---|
| Formaldehyde | Motor vehicles, photochemistry | Furniture, particle boards |
| Benzene | Motor vehicles, industry | Smoking, ETS |
| Benzo[a]pyrene | Coke/aluminum plants, diesel vehicles | Smoking, ETS |
| Tetrachloroethylene | Dry cleaning shops | Dry-cleaned clothes |
| Chloroform | Sewage treatment plants | Taking showers |
| p-Dichlorbenzene | Chemical manufacturing | Air deodorizers |
| Particulate matter | Industry, motor vehicles, photochemistry | Smoking, ETS, unvented combustion devices (indoors) |
| Carbon monoxide | Motor vehicles | Driving, gas stoves |
| Nitrogen dioxide | Motor vehicles, industry | Gas stoves |

50 g of PM per person was released into the atmosphere as environmental tobacco smoke (ETS) based on the average 2,080 cigarettes smoked annually per capita. Thus PM mass from ETS was only 3% of that from power plants. Since power plant emissions were released high in the atmosphere away from populated areas, whereas ETS occurs indoors and was breathed by many people for many hours, relative exposure to PM from ETS was about 80 times higher than from power plants[14]. Similar findings have been derived from many other studies of indoor air quality or those of human exposures around the world, indicating that major emission sources and exposure sources can be quite different for a large number of toxic air pollutants[13,32,33,34,35,36]. Examples are shown in Table 1.

These findings of emissions vs. exposures comparisons have stimulated, in the past decade or so, the development of simple approaches for linking source emissions and population exposure for use in risk and decision analysis of air pollution[14,37]. Perhaps the simplest approach is the use of a term called exposure effectiveness or exposure efficiency[14,38]. The term is defined as the fraction of material released from a source that is actually exposed (inhaled) by a population of concern. For example, Smith examined major PM sources worldwide and estimated exposure effectiveness for these sources (see Table 2)[14]. The availability of exposure effectiveness data can be used to support the need for switching the conventional emission-based framework to the exposure-based framework of ranking of sources. This has important implications in developing most cost-effective control strategies. However, the fate and transport of pollutants from their emission sources to exposure receptors can be very complex, making the determination of exposure effectiveness very challenging and difficult [39]. Therefore, mechanistic and probabilistic models to link emissions to population exposure should be used, in addition to the use of exposure effectiveness values, to reduce uncertainty for overall policy analysis.

## ASSESSING HUMAN EXPOSURE TO AIR POLLUTANTS

As shown in Fig. 1, exposure is an important component of the air pollution system and is at the middle point of the continuum, linking the source and the health endpoints. Exposure assessment is considered one of the four key components in the heath risk assessment paradigm commonly accepted today[40]. Risk assessment provides scientific bases for risk management decisions on priority setting, determination of unacceptable risks, selection of cost-effective approaches to

**TABLE 2**
**Exposure Effectiveness for Airborne Particulate Matter by Source Type**

| Source | Exposure Effectiveness |
|---|---|
| Active cigarette smoking | 0.8* |
| Environmental tobacco smoke | $3 \times 10^{-3}$ |
| Domestic stove (unvented) | $3 \times 10^{-3}$ |
| Domestic stove (vented outdoors) | $2 \times 10^{-3}$ |
| Neighborhood sources | $4 \times 10^{-5}$ |
| Motor vehicles | $2 \times 10^{-5}$ |
| Power plants (developing countries) | $1 \times 10^{-5}$ |
| Coal-fired power plants (U.S.) | $1 \times 10^{-6}$ |

*Assuming 80% of particles from cigarette smoking are inhaled by the smoker. Adapted from [14].

preventing or reducing risks, and evaluation of the effectiveness of risk mitigation efforts[41,42]. Exposure data are crucial to each of these decisions, as shown in the examples above section[43].

Measurement or estimation of exposure is essential in environmental epidemiologic studies. In the past decade or so, a large number of epidemiologic studies have been conducted worldwide to examine associations between exposures to outdoor PM (and other air pollutants in some studies) and various health endpoints (e.g., mortality rates, morbidity prevalence, and pulmonary function)[44,45]. The findings of these studies are very useful in evaluating, and revising when necessary, current air pollution standards that are set to protect the public health. The excess risk for mortality and morbidity rates, associated with a realistic maximum incremental change in pollutant concentrations, is usually small (even when statistically significant) with relative risks or odds ratios typically less than 2. The low-level risks require that exposure estimates must be fairly accurate and reliable, otherwise the presence of many confounding factors could affect the accuracy and usefulness of the results. Despite the progresses made in improving exposure estimates in a number of published studies, misclassification or inaccurate quantification of exposures are still recognized as one of the major error/uncertainty sources of the existing data on health effects of ambient PM[45]. The need for more accurate and robust exposure estimates will certainly promote the advancement in methodologies and techniques of exposure assessment.

Exposures to air pollutants, and other types of hazardous agents, can be estimated qualitatively or quantitatively, depending upon the purpose of exposure assessment and the availability of relevant data. Questionnaires have been widely used in exposure studies and epidemiologic studies[46,47,48,49]. In exposure studies, measurements of pollutant concentrations are often accompanied by questionnaire surveys. Incorporating concentration data and questionnaire data can significantly reduce uncertainties of exposure estimates. However, many epidemiologic studies have relied solely on the questionnaire approach to obtain exposure estimates. In these studies, questionnaire respondents or other subjects of concern are typically categorized into two or more groups (e.g., exposed/unexposed, highly exposed/moderately exposed/unexposed). Such a type of qualitative and retrospective method, for example, has been employed to estimate study subjects' exposure to indoor air pollution in a number of air pollution epidemiologic studies[46,50,51,52]. Exposures have been characterized as to whether certain types of pollution sources (e.g., active or passive tobacco smoking, gas stoves, coal stoves, domestic sources of allergens, outdoor pollution sources) were present in the residences of study subjects[46,53]. This type of exposure assessment is perhaps the simplest and cheapest method and is especially useful in studies involving a large number of human subjects. Obviously, the method depends on a prior knowledge of exposure and their determinants to develop effective questionnaires. The accuracy of information obtained from questionnaire surveys can be negatively impacted by both biased questionnare design and subjective responses to the questions. Quantitative estimation of exposure, when possible, can improve the quality of epidemiologic analysis.

The quantitative estimation of exposure is often required in risk assessment and relevant policy analysis. Quantifying exposure to air pollutants essentially requires the measurement or the estimation of pollutant concentrations and coupling concentration data with the length of contact by an individual. Measuring or predicting atmospheric concentrations of air pollutants has been the central theme throughout the history of assessing air quality for air pollution. When this is put in the context of exposure, however, the concentrations need to be measured at, or as close as possible to, the point of contact with the human body[27]. In other words, exposure measurement at individual levels requires measuring exposure concentration, which is defined as the concentration of a pollutant in the carrier medium at the contact boundary of the body[18,28]. However, many epidemiologic studies are specifically interested in the investigation of health effects of population or community exposure to ambient (outdoor) air pollutants. In this case, it is more important and necessary to develop appropriate surrogates for exposure to ambient pollutants than to assess total personal (individual) exposure[54,55,56]. For example, historical data from fixed monitoring stations have been used to reasonably address public health effects of exposure to ambient air pollutants. This is achieved with sophisticated statistical approaches to sort out “average” community (population) exposure from inter-individual exposure variations in both cross-sectional and cohort studies[50,51,52,57].

Direct measurements of exposure concentrations, also known as point-of-contact measurements, quantify exposure while it is occurring[21]. This type of measurement has been often done using personal dosimeters or personal monitors. Personal monitoring requires that subjects transport the sample collection/monitoring devices with them at all times during the assessment period. Small and lightweight monitors with sufficient analytical sensentivity have made personal monitoring possible and practical. Personal monitors can be grouped into two general categories: integrated samplers that collect the pollutant over a specified time period and then are returned to the laboratory for subsequent analysis, and continuous monitors that use self-contained analytical systems to measure and record the pollutant concentration on the spot[58,59]. Either of the categories can be active samplers or passive samplers. An active sampler uses a pump and a power source to pull air passing a collecting medium or a sensor. A passive sampler depends on molecular diffusion to bring the pollutant molecules into contact with a collecting medium or a sensor. Examples of personal samplers/monitors, which are capable to measure typical ambent levels of pollutants, are shown in Table 3. The major strength of this approach is the direct measurement of exposure during the monitoring period. Due to logistic constraints, this time period is typically short, thus limiting the usefulness of its value in estimating long-term exposure. Direct measurement of exposure concentrations is costly and time consuming for large population studies, can be burdensome for study participants, and is often constrained by lack of suitable analytical methods for many pollutants[12,18,20].

When direct measurements are not available or not practical, the so-called scenario construction approach is often used to estimate exposures. Commonly this is done by merging two essential components of exposure: concentration in a specific microenvironment (e.g., outdoors, indoors, in transit) and contact duration in the microenvironment. The integrated exposure can be estimated as the sum of exposures occuring in each microenvironment. The mathematical application of this approach enables the simplification of Eq. 1 to Eq.3.

**TABLE 3**
**Personal Samplers/Monitors Employed in Environmental Exposure Measurements***

| Monitor Type | Pollutant | Collection Mechanism | Analytical Method |
|---|---|---|---|
| Integrated, active | Respiratory particles (e.g., $PM_{10}$, $PM_{2.5}$) | Pump, filter (Teflon filter, quartz fiber filter, etc.) | Gravimetric for mass concentrations; IC for ionic constituents (sulfate, nitrate, ammonium, etc.); XRF or ICP-MS for elements; GC-MS for PAHs; thermal-optical techniques for elemental carbon/organic carbon |
| | Nonpolar VOCs | Pump, Tenax cartridge | Thermal desorption, GC-MS or GC-FID |
| | Organochlorine pesticides | Pump, polyurethane foam, | Solvent extraction, GC-ECD |
| Integrated, passive | VOCs | Diffusion, filter impregnated with activated carbon | Solvent extraction, GC-FID or GC-MS |
| | Nitrogen dioxide | Diffusion, TEA coated adsorbent | Solvent extraction, IC |
| | Nitrogen dioxide | Diffusion, filter pad | Colorimetric - Saltzman reagent |
| | Sulfur dioxide | Diffusion, filter pad | Solvent extraction, IC or colorimetric |
| | Ozone | Diffusion, nitrite coated filter | Solvent extraction, IC |
| | Formaldehyde | Permeable membrane, MBTH | Pararosaniline reaction, colorimetric |
| | Aldehydes and ketones | Diffusion badge or tube, DNPH impregnated sorbent | Solvent extraction, HPLC-UV |
| | Aldehydes and ketones | Diffusion tube, DNSH impregnated sorbent | Solvent extraction, HPLC-fluorescence |
| | Polynuclear aromatics | Diffusion badge, sorbent | Phosphorescence |
| Continuous, active | Carbon monoxide | Pump, electrolyte | Electrochemical |
| | Particles | Pump, sensing chamber | Light scattering |

* Those designed primarily for occupational settings are not included in the table.
*Note*: IC = ion chromatography; GC = gas chromatography; MS = mass spectrometry; FID = flame ionization detector; ECD = electron capture detector; XRF = X-ray fluorescence; ICP = induced coupled plasma spectrometry; PAHs = polyaromatic hydrocarbons; TEA = triethanolamine; MBTH = 3-methyl-2-benzothiozolinone; DNPH = 2,4-dinitrophenylhydrozine; HPLC = high pressure (performance) liquid chromatography; DNSH = 5-dimethylaminonaphthalene-1-sulfohydrazide.

$$E = \sum_{i=1}^{n} C_i T_i \qquad (3)$$

where $C_i$ is the average pollutant concentration for the $i^{th}$ microenvironment where an individual or a population of concern has spent a time duration of $T_i$, and $n$ is the total number of microenvironments encountered by the individual or the population over the specified time period of concern[17,20,21]. Microenvironmental concentrations can be measured using fixed-site (stationary) monitoring or estimated using source-pathway-receptor modeling. Because fixed-site monitoring typically involves measurements of concentrations in homes, the samplers have to generate low-level noise. These types of measurements are usually done on a 24- or 48-h basis to represent daily exposures[60,61]. Passive samplers/monitors can be ideal for microenvironmental measurements to measure daily or weekly exposures[62,63,64,65]. Pollutant concentrations in microenvironments can be determined through mathematical modeling of pollutant fate and transport processes from emission sources to the concerned microenvironments[66,67,68]. Recently, a few exposure modeling frameworks have been developed incorporating existing source/receptor/pharmacokinetics models[69,70,71,72]. Exposure duration in each concerned microenvironment can be estimated using exisiting data and knowledge with reasonable assumptions, or by collecting specific data, about time-activity patterns, lifestyle characteristics, and other relevant factors[21,49]. The primary advantage of this approach is that it enables making exposure estimation with very limited data. Incorporating historical air pollution data and time-activity data allows exposure assessors to make exposure estimates retrospectively, which is desirable for retrospective epidemiologic studies of health effects of past exposure. Although conceptually sound and mathematically sophisticated, these source-to-dose modeling frameworks typically involve a large number of assumptions that can potentially generate large uncertainties. For example, the number of microenvironments considered may often be smaller than the number of microenvironments encountered by the individuals or populations of concern, thus leading to inadequate characterization of exposure. Moreover, the pharmacokinetic/pharmacodynamic data on many important air pollutants such as PM are lacking or poor in data quality. The validity of these modeling frameworks for predicting population exposures remains to be evaluated in future epidemiologic analyses and health risk assessment.

Lastly, dose information obtained via the use of biological markers linked with pharmacokinetic models can be used to estimate exposure[21,27]. If measurements of body burden or elimination levels are available, exposures leading to such body burden or elimination levels can be estimated by incorporating information or assumptions about rates of intake and uptake. Appropriate use of this approach depends on the availability of biological markers in human tissues or fluids (e.g., adipose tissue, blood, bone, breast milk, breath, feces, hair, urine, teeth) and adequate information for estimating intake, uptake, and metabolic rates[21,73,74]. There are a number of advantages to this approach. The approach can demonstrate that exposure and uptake have occurred. It measures internal dose, which is more directly – compared to exposure – associated with health effects. However, since internal dose is integrated across all routes of exposure, the approach does not usually provide information about the relative importance of inhalation, ingestion, and dermal exposure, thereby limiting the usefulness for setting exposure reduction strategies[75]. For most air pollutants, there is a lack of understanding of pharmacokinetics/pharmacodynamics including bioavailability, absorption, metabolism, and elimination. This has been a major obstacle of implementing the biomonitoring method in exposure assessment.

Under some circumstances, air pollution exposure assessment may need to take into consideration other routes of exposure when air pollutants are transported from the atmosphere to other environmental media (e.g., water, soil) before reaching people's breathing zones. It is well known that atmospheric deposition is the ultimate pathway by which gaseous and particulate pollutants are removed from the atmosphere. People may have a chance to contact the soils and waters (surface water or groundwater) containing the pollutants transported from the atmosphere[15,76]. In this case, assessing exposure resulting from ingesting contaminated water and soil can be an important part of analysis of the total risk associated with the air pollution.

The use of several innovative techniques has been reported in the recent literature to estimate exposure or help reduce estimation uncertainty. Satellite-based remote sensing has been used to reconstruct historical source patterns or to track travel patterns of human subjects[77,78]. In a community-based urban air toxics study conducted in Oklahoma, global positioning system (GPS) technology has been used to automatically log subjects' locations as the subjects went about their daily activities[78]. During the study, the subjects wore GPS data recorders that tracked the subjects' movements on trips outside the immediate vicinity of their homes. A

comparison of the GPS data with time-activity diaries kept by the subjects indicates that the GPS data confirmed all travel events in almost all cases, and furthermore, detected some missing travel events not recorded in the diaries. Given that one of the largest uncertainty sources of exposure assessment is the accuracy of time-activity data, the GPS technique may prove to be a promising means in future studies involving the collection of time-activity data. However, the current GPS technique needs to be improved to overcome a number of limitations including the limited capacity of GPS recorders and blocking of GPS satellite transmissions by steel-reinforced structures, vehicle body panels, and other electrically conductive media[78].

The use of geographic information systems (GIS) has emerged in recent studies involving the assessment of individual exposure and population exposure[79,80,81]. In these studies, historical exposure to components (e.g., $NO_2$, $SO_2$, and PM) of ambient air pollution has been estimated using emission data, dispersion models, and GIS data. The use of GIS can enable a more accurate estimate of population exposure and a more effective control for confounding effects in population-based case-control studies of air pollution health effects[80,81]. For example, in the Small Area Variations in Air Pollution and Health Study (SAVIAH), the researchers used GIS that allowed them to estimate the study children's exposure to ambient $NO_2$ and $SO_2$ pollution in four levels: (1) level of ecological exposure, estimated for each geographical area as the means of calculated $NO_2$ and $SO_2$ levels at all the study sites; (2) level of individuals' school exposure, estimated from average concentrations of $NO_2$ and $SO_2$ in "buffer" areas around the schools; (3) level of individuals' home exposure, estimated from average concentrations of $NO_2$ and $SO_2$ in "buffer" areas around children's residences; and (4) level of mean individual exposure, estimated from the average of home and school exposures. This type of stratification in exposure estimates enabled the researchers to address the effects of ambient $NO_2$ and $SO_2$ pollution at both an ecological level and individual level[80]. (The subjects' exposure to indoor pollution sources such as gas cooking and other potential risk factors, estimated using the information from the questionnaire survey, were controlled in the models of epidemiologic analysis.) The use of GIS can also be helpful in assessing inter- and intra-individual (through longitudinal data) sources of variation in exposure to airborne pollutants. Understanding inter- and intra-individual variability can reduce uncertainties in exposure estimates and thus improve the results of epidemiological studies and of risk assessment in policy analyses[82]. This can be achieved by using most appropriate study designs and data analysis methods.

## CONCLUDING COMMENTS

Emissions of major industrial sources have been regulated for hazardous air pollutants (HAPs), also called air toxics, in the U.S. and some other countries. The regulations, in theory, are based on the toxicity of regulated pollutants or the chemical reactivity of regulated pollutants in generating secondary toxic pollutants. Some of these HAPs (e.g., benzene, formaldehyde) can be emitted from nonindustrial sources and may be present at occupational and residential settings. The largest fraction of human exposure to these HAPs may result from nonregulated sources. This has raised questions about the effectiveness of the current HAPs reduction strategy[11,12,31]. In theory, health risk assessment constitutes part of the basis for governmental action on environmental problems in the U.S. and other countries. The current U.S. environmental regulations for carcinogens commonly aim to limit the individual lifetime risk of premature death to $\sim10^{-6}$–$10^{-5}$ for contaminants in drinking water and outdoor air. However, average lifetime risks of premature death from exposure to indoor air pollutants are at least $\sim10^{-4}$ to $10^{-3}$, and maximum individual risks may exceed $10^{-2}$[83]. In a recent editorial in *Indoor Air*, Nazaroff and Weschler raised a flagpole question: "Does it make sense to spend large sums to mitigate environmental risks at a hazardous waste site of $10^{-6}$ when indoor air quality risks remain unchecked in the range of $10^{-4}$ to $10^{-2}$?"[83].

This by no means implies that regulations on large industrial and mobile emission sources are not necessary. Indeed, these regulations have played a vital rule in air pollution history. For example, the implementation of these regulations in developed countries has prevented severe smog episodes. In the U.S., the success in acid rain mitigation is attributable to the reduction in emissions of $SO_2$ and $NO_x$ from regulated large industrial sources and residential oil burning sources. Although $O_3$ concentrations in many urban areas of developed countries are often above the current ambient air quality standards, the situation would be much worse without regulating stationary and mobile sources of $O_3$ precursors (hydrocarbons and $NO_x$)[1,10,84]. In some cases, it is necessary to regulate sources from a standpoint of climate impact, ecological impact, or aesthetic impact[15,85]. Hence, exposure-oriented monitoring and exposure-effectiveness calculations should not be interpreted simply as substitutes for ambient monitoring, emissions inventories, and other traditional tools. There is no doubt, however, that exposure-based regulations have a greater potential for increasing the cost effectiveness of control strategies, especially when the resources for mitigation are limited[86].

Exposure analysis has been a fast-growing scientific field in the last 2 decades and has an even greater potential to grow[30]. Future air pollution problems will be more complex as they will have fewer characteristics of historical smog episodes; more likely they will appear as complex mixtures of multiple pollutants present at lower levels, and they will produce more subtle health effects. We certainly need more sophisticated and integrated approaches, such as risk management, to effectively deal with more complex problems. Achieving accurate exposure estimates is a critical step toward successful application of integrated approaches[43]. Fostering the growth of exposure assessment sciences requires improvement of current methods and development of new and innovative methods that can be used for better risk assessment, better environmental epidemiology, better environmental toxicology, and better source/receptor/effects modeling[87]. For example, diffusive samplers need to be developed for many toxic gaseous pollutants that cannot be effectively measured with the point-of-contact approach using current technologies. Currently available active samplers need to be improved to have lighter weight, lower noise, and longer battery life. This is possible with quickly developing battery and electronic technologies. Developing and utilizing appropriate mathematical models to estimate human exposure will be more desirable as capacities of collecting data (e.g., GIS data, census data, air pollution monitoring data) increase and commercially available statistical tools (e.g., SAS, SPSS) advance[78,88]. Rapid advancements in biological sciences (e.g., molecular biology and genomics) can shed new lights and perhaps lead to a breakthrough in the development of molecular markers to assess exposure.

## ACKNOWLEDGEMENTS

The assistance of Mr. Robert C. Harrington in organizing references is highly appreciated. Work of Drs. J. Zhang and P. Lioy is supported in part by NIEHS Center Grant 05022-10.

## BIOSKETCHES

**Junfeng (Jim) Zhang**, Ph.D., is Director, EOHSI Center for International Health, Piscataway, NJ, and Associate Professor, Environmental and Occupational Health Division, University of Medicine and Dentistry of New Jersey - School of Public Health. Prof. Zhang received his Ph.D. degree (1994) in Environmental Science and Public Health from Rutgers University and UMDNJ, an M.S. degree (1991) in Environmental Sciences from Rutgers University, an M.S. degree (1988) in Atmospheric Chemistry from Peking University, and a B.S. degree (1985) in Applied Chemistry from Peking University. His professional experience includes the following: 1987–1989 Consultant, Environmental Protection Office of Peking University, Beijing, China; 1988–1989 Executive Director of Management Training Program, Stone Company, Beijing, China; 1989–1994 Research Assistant, Environmental and Occupational Health Sciences Institute; 1990 Air Pollution Analyst, Gibbs & Hill, Inc., NY; 1992 Analytical Chemist, Chicopee Research, Johnson & Johnson, Inc., NJ; 1994–1995 Fellow, Program on Environment, East-West Center, Honolulu, Hawaii; 1995–Present, Member, Exposure Measurement and Assessment Division, Environmental and Occupational Health Sciences Institute, jointly sponsored by University of Medicine and Dentistry of NJ (UMDNJ) and Rutgers University; 1995–2001; Assistant Professor, Department of Environmental and Community Medicine, UMDNJ-Robert Wood Johnson Medical School; 1996–Present, Graduate Faculty, Department of Environmental Sciences, Rutgers, The State University of NJ; 2000–2001 Lecturer, TH Huxley School of Environment, Earth Science and Engineering, Imperial College of Science, Technology and Medicine, London, UK; 2000–Present, Adjunct Research Professor, China National Environmental Monitoring Center, Beijing, China; 2001–Present, Director, EOHSI Center for International Health, Piscataway, NJ; 2001–Present, Associate Professor, Environmental and Occupational Health Division, UMDNJ - School of Public Health. Prof. Zhang is a member of the International Society of Exposure Analysis; Air and Waste Management Association; American Chemical Society; and American Industrial Hygiene Association. He has won numerous awards, including: 1985-Outstanding Graduate (Highest Honors), Peking University; 1990-Second Place, National Best Books on Environmental Knowledge, Beijing, China, A Treasure Box of Environmental Knowledge; 1992-First Place, National Hongyu (Rainbow and Rain) Cup Contest of Poetry, Beijing, Memory; 1993-Second Place, Contest of Student Paper Session, 39$^{th}$ Anniversary Conference of Air & Waste Management Association, Mid-Atlantic States Section, Atlantic City, NJ. In 1998 he was Invited site-visit team member, Engineering Research Center (ERC), National Science Foundation (NSF). In 1999 he was on the Airborne Particulate Matter Health Effects (STAR

Research Grants) Panel, Washington, DC, National Center for Environmental Research and Quality Assurance, U.S. EPA and was an Invited Expert for IPCC's Expert Group Meeting on Greenhouse Gases Emission Inventories - Energy Sector, Prague, Czech Republic.

**Paul J. Lioy,** Ph.D., is Professor, New Jersey School of Public Health, University of Medicine and Dentistry of New Jersey, and Acting Associate Director, Environmental and Occupational Health Sciences Institute. Prof. Lioy received his Ph.D. (1975) and M.S. (1973) degrees in Environmental Science from Rutgers University, an M.S. degree (1971) in Physics and Applied Math from Auburn University, and a B.A. degree (1969) in Physics and Education from Montclair State College. His professional experience includes the following: 1976–1978 Lecturer, Department of Civil Environmental Engineering, Polytechnic Institute of New York; 1978–1982 Assistant Professor, Institute of Environmental Medicine NYU Medical Center; 1982–1985 Associate Professor, Institute of Environmental Medicine, NYU Medical Center; 1985–1989 Associate Professor, Department of Environmental and Community Medicine, UMDNJ-Robert Wood Johnson Medical School, Piscataway; 1986–Present, Graduate Faculty of Rutgers University, Department of Environmental Science, Toxicology Program; 1986–Present, Director, Exposure Measurement and Assessment Division, EOHSI, UMDNJ and Rutgers University; 1986–Present, Chief EMAD, ECM, UMDNJ-RWJMS; 1989–Present, Professor, Department of Environmental and Community Medicine UMDNJ-Robert Wood Johnson Medical School; 1995–2001 Deputy Director, EOHSI of UMDNJ-RWJMS, and Rutgers University, Piscataway; 2001–Present, Professor, New Jersey School of Public Health-UMDNJ, and Acting Associate Director, EOHSI.
